# Positive Associations of Serum Concentration of Polychlorinated Biphenyls or Organochlorine Pesticides with Self-Reported Arthritis, Especially Rheumatoid Type, in Women

**DOI:** 10.1289/ehp.9887

**Published:** 2007-02-20

**Authors:** Duk-Hee Lee, Michael Steffes, David R. Jacobs

**Affiliations:** 1 Department of Preventive Medicine and Health Promotion Research Center, School of Medicine, Kyungpook National University, Daegu, Korea; 2 Department of Laboratory Medicine and Pathology, University of Minnesota, Minneapolis, Minnesota, USA; 3 Division of Epidemiology, School of Public Health, University of Minnesota, Minneapolis, Minnesota, USA; 4 Department of Nutrition, University of Oslo, Oslo, Norway

**Keywords:** arthritis, persistent organic pollutants, pesticides, polychlorinated biphenyls, rheumatoid arthritis

## Abstract

**Background:**

Persistent organic pollutants (POPs) can influence the immune system, possibly increasing the risk of rheumatoid arthritis (RA). In addition, as metabolic change due to obesity has been proposed as one mechanism of osteoarthritis (OA), POPs stored in adipose tissue may be also associated with OA.

**Objective:**

Our goal in this study was to examine associations of background exposure to POPs with arthritis among the general population.

**Design:**

We investigated cross-sectional associations of serum POPs concentrations with the prevalence of self-reported arthritis in 1,721 adults ≥ 20 years of age in the National Health and Nutrition Examination Survey 1999–2002.

**Results:**

Among several POPs, dioxin-like polychlorinated biphenyls (PCBs) or nondioxin-like PCBs were positively associated with arthritis in women. After adjusting for possible confounders, odds ratios (ORs) were 1.0, 2.1, 3.5, and 2.9 across quartiles of dioxin-like PCBs (*p* for trend = 0.02). Corresponding figures for nondioxin-like PCBs were 1.0, 1.6, 2.6, and 2.5 (*p* for trend = 0.02). Organochlorine (OC) pesticides were also weakly associated with arthritis in women. For subtypes of arthritis, respectively, RA was more strongly associated with PCBs than was OA. The adjusted ORs for RA were 1.0, 7.6, 6.1, and 8.5 for dioxin-like PCBs (*p* for trend = 0.05), 1.0, 2.2, 4.4, and 5.4 for nondioxin-like PCBs (*p* for trend < 0.01), and 1.0, 2.8, 2.7, and 3.5 for OC pesticides (*p* for trend = 0.15). POPs in men did not show any clear relation with arthritis.

**Conclusions:**

The possibility that background exposure to PCBs may be involved in pathogenesis of arthritis, especially RA, in women should be investigated in prospective studies.

Persistent organic pollutants (POPs) are organic chemical compounds that are highly toxic, persist in the environment, bioaccumulate in fatty tissues of living organisms, travel long distances, and naturally flow toward colder climates (Abelsohn et al. 2002). Humans are generally exposed to POPs through their food supply (Abelsohn et al. 2002).

Whether the exposure to endocrine disruptors such as POPs at current background environmental levels is harmful to human health has become a matter of intense debate, politically and scientifically (Kaiser 2000). However, we recently reported striking associations of serum concentrations of several POPs with diabetes in a random sample of the general population with background exposure to POPs (Lee et al. 2006b). In a recent editorial (Lee et al. 2006a), we discussed that selection of a reference group with a known very low exposure is critical in the estimation of POPs-associated risks. This is because risks of several POPs-associated conditions appear to increase substantially even within a narrow range of low POPs concentrations, not detectable without substantial blood volume. Thus, epidemiologic studies on POPs in the general population could identify strong associations that might have been missed in previous epidemiologic studies in people exposed to high concentrations of selected POPs that used the general population as the reference group, as if its substantial range of exposure had uniform risk.

In the present study we hypothesized that background environmental exposure to POPs is also involved in pathogenesis of arthritis. Among the various subtypes of arthritis, rheumatoid arthritis (RA) and osteoarthritis (OA) are the two most common in the general population (Abyad and Boyer 1992). RA is an autoimmune disease in which an as yet unknown trigger results in a chronic inflammatory process affecting the synovial membrane of the joints (Gabriel 2001), while OA is often thought to result from natural aging processes on the joint surfaces (Sharma et al. 2006). Although much is known about the pathophysiology of these conditions at a cellular level, there is considerably less information about the etiology of RA and OA in general population studies.

Endocrine disruptors such as POPs markedly influence the immune system (Ahmed 2000), which could increase the risk of autoimmune diseases such as RA. In addition, beyond the negative effects of increased weight bearing caused by obesity, metabolic change due to adipose tissue has recently been proposed as one underlying mechanism of osteoarthritis (Dumond et al. 2003). Because POPs stored in adipose tissue can be related to differentiation, metabolism, and function in adipose tissue (Mullerova and Kopecky 2006), POPs could be involved in the relation between obesity and OA. Furthermore, the relations of POPs with arthritis may be different depending on sex because endocrine disruptors such as POPs may exert their effects through sex hormone-related receptors (Crews et al. 2000).

The population-based National Health and Nutrition Examination Survey (NHANES) 1999–2002 measured background concentrations of a variety of POPs. This survey also obtained self-report of clinical diagnosis of history of arthritis and subtypes of arthritis. Although the validity of report of all types of arthritis combined is high, validity of the subtype of arthritis based on questionnaire has been reported to be low (Star et al. 1996). Given this fact, the predominance of RA and OA among all arthritis types, and our hypothesis that jointly involved RA and OA, we primarily focused our investigation on the association between serum concentrations of POPs and prevalence of all arthritis and further analyzed by the subtype of arthritis.

## Materials and Methods

The 1999–2002 NHANES conducted by the National Center for Health Statistics (NCHS) of the Centers for Disease Control and Prevention (CDC) was designed to be nationally representative of the noninstitutionalized U.S. civilian population on the basis of a complex, multistage probability sample. Details of the NHANES protocol and all testing procedures are available elsewhere (NCHS 2006a, 2006b). Serum concentrations of biologically important POPs or their metabolites were measured in subsamples of the NHANES 1999–2002 (NCHS 2005). The study protocol was reviewed and approved by the CDC institutional review board; additionally, informed written consent was obtained from all subjects before they took part in the study.

The NHANES standardized home interview was followed by a detailed physical examination in a mobile evaluation clinic or the participant’s home (NCHS 2006a, 2006b). Information about existing medical conditions was collected using questionnaires. Venous blood samples were collected and shipped weekly at –20°C. POPs were measured by high-resolution gas chromatography/high-resolution mass spectrometry using isotope dilution for quantification. All of these analytes were measured in approximately 5 mL serum using a modification of the method of Turner et al. (1997). Ability to detect low POPs concentrations was greater in those participants who provided a larger aliquot. The POPs were reported on a lipid-adjusted basis using concentrations of serum total cholesterol and triglycerides.

Although 49 POPs were measured in both NHANES 1999–2000 and 2001–2002, to avoid bias in estimation among those below the limit of detection (LOD) we selected the 19 POPs for which at least 60% of study subjects had concentrations > LOD; 3 polychlorinated dibenzo-*p*-dioxins (PCDDs), 3 polychlorinated dibenzofurans (PCDFs), 4 dioxin-like polychlorinated biphenyls (PCBs), 5 nondioxin-like PCBs, and 4 organochlorine (OC) pesticides. A total of 1,721 study participants were ≥ 20 years of age with information available on serum concentrations of the 19 selected POPs.

For each POP, subjects with serum concentrations < LOD were regarded as the reference group, and subjects with detectable values were categorized into quartiles by cutoff points of 25th, 50th, and 75th values. To yield a cumulative measure (which would provide a cumulation of risk across POPs with similar chemical and physical properties) of 3 PCDDs, we summed the rank of 3 POPs that belong to the PCDDs. The summary values were categorized into quartiles by cutoff points of 25th, 50th, and 75th values. We categorized and cumulated POP subclasses similarly for the 3 PCDFs, the 4 dioxin-like PCBs, the 5 non-dioxin-like PCBs, and the 4 OC pesticides. For example, a subject had rank 0 for all POPs with serum concentrations < LOD. For other POPs with a detectable level, the participant was ranked accordingly. Thus, depending on the sum of ranks of the several POPs belonging to the specific POP subclass under consideration, the subject could be in the lowest quartile or in a higher quartile; however, if all POPs in the subclass were nondetectable, the subject would be placed in the lowest quartile. We chose the current approach because there is no scientific rationale for summing within the POP subclasses to create broader exposure categories.

Participants were considered to have prevalent arthritis if they answered “yes” to the following question: “Has a doctor or other health professional ever told you that you had arthritis?” They were further asked about the type of arthritis. We used logistic regression models to calculate multivariate-adjusted odd ratios (ORs) and 95% confidence intervals (CIs). All analyses were performed separately in men and women. Adjusting variables were age (years), race/ethnicity, poverty income ratio (continuous), body mass index (BMI; continuous), and cigarette smoking (never, former, or current). We substituted median values of noncases for missing BMI or poverty income ratio in 83 subjects; exclusion of these individuals did not change any conclusions.

We performed all statistical analyses with SAS 9.1 (SAS Institute Inc., Cary, NC, USA) and SUDAAN 9.0 (Research Triangle Institute, Research Triangle Park, NC, USA). Estimates of main results were calculated accounting for stratification and clustering (Korn and Graubard 1991), adjusting for age, race and ethnicity, and poverty income ratio instead of using sample weights; this adjustment is a good compromise between efficiency and bias (Graubard and Korn 1999; Korn and Graubard 1991). Because results were very similar with SAS 9.1 and SUDAAN 9.0, we present the results based on SAS 9.1.

## Results

The sample of 1,721 participants was 44.7% male, 47.7% white, and 16.9% current smokers. Mean ± SD for age was 49.2 ± 19.0 years (range 20–85 years). Table 1 shows the associations of five subclasses of POPs with demographic or health behavior factors. Age was the strongest and most important correlate of serum concentrations of all five subclasses of POPs in both sexes, with correlation coefficients ranging from 0.35 to 0.72. White subjects had lower concentrations of OC pesticides in both sexes and lower PCDDs in females, but higher concentrations of PCDFs and PCBs. Those with higher income had lower concentrations of OC pesticides but higher PCBs. Males with higher BMI tended to have higher concentrations of most POPs; however BMI was not associated with POPs among females, except in the inverse association with nondioxin-like PCBs. Current smokers tended to have lower concentrations of most POPs. After adjusting for age, we found positive pairwise correlations among serum concentrations of the five subclasses of POPs with correlation coefficients from 0.24 to 0.73 in men and 0.19 to 0.77 in women. Women had higher serum concentrations of all five subclasses of POPs with age-adjusted correlation coefficients with sex of 0.22–0.34.

There were 414 prevalent self-reported arthritis cases (164 men and 250 women): 93 RA, 116 OA, 37 other types of arthritis, and 168 unspecified arthritis cases. Neither PCDDs nor PCDFs were associated with arthritis in either sex. However, women who had higher concentrations of dioxin-like PCBs or nondioxin-like PCBs showed a higher risk of prevalence of arthritis (Table 2). After adjusting for age, race/ethnicity, poverty income ratio, BMI, and smoking, ORs were 1.0, 2.1, 3.5, and 2.9 across quartiles of dioxin-like PCBs (*p* for trend = 0.02). Corresponding figures for nondioxin-like PCBs were 1.0, 1.6, 2.6, and 2.5 (*p* for trend = 0.02). OC pesticides were weakly associated with the prevalence of arthritis among women; adjusted ORs were 1.0, 1.2, 1.3, and 1.8 (*p* for trend = 0.09) (Table 2). We also performed analyses adjusting for sex (males and females in one model); adjusted ORs for dioxin-like PCBs were 1.0, 1.6, 2.1, and 2.0 (*p* for trend = 0.02) and those for nondioxin-like PCBs were 1.0, 1.4, 3.0, and 2.1 (*p* for trend < 0.01). However, we found little association among males (Table 2), and we observed significant *p*-values for sex interaction: 0.03 for dioxin-like PCBs and 0.04 for nondioxin-like PCBs. When the study subjects were stratified by age (< 50 and ≥ 50 years) or obesity (BMI < 30 and ≥ 30), the associations were similar to those in Table 2. Most specific POPs belonging to the subclasses of dioxin-like and nondioxin-like PCBs and OC pesticides were positively associated with arthritis among women (Table 3).

For subtypes of arthritis among women, RA (*n* = 93) was more strongly associated with dioxin-like PCBs, nondioxin-like PCBs, or OC pesticides than was OA (*n* = 116) (Table 4). The adjusted ORs (by quartile) for RA were 1.0, 7.6, 6.1, and 8.5 for dioxin-like PCBs (*p* for trend = 0.05), 1.0, 2.2, 4.4, and 5.4 for nondioxin-like PCBs (*p* for trend < 0.01), and 1.0, 2.8, 2.7, and 3.5 for OC pesticides (*p* for trend = 0.15). Adjusted ORs for unspecified arthritis subtype (*n* = 168) were weaker than those of RA but stronger than those of OA (Table 4), as we expected because these cases were likely a mixture of mostly RA and OA.

In all analyses, we also considered possible confounding by self-reported weight loss in the 1 year or in the 10 years before examination, because weight loss has been reported to increase serum concentrations of POPs (Chevrier et al. 2000). However, the adjustment for weight loss did not materially change the results (data not shown). Additionally, we investigated the associations after excluding subjects with diabetes or cardiovascular diseases, but the results were not different (data not shown).

## Discussion

In the present study, background exposure to some kinds of POPs was positively associated with arthritis among women. Especially, among the two most common subtypes of arthritis, RA showed much stronger associations with POPs than did OA, and those associations were of intermediate strength in those with unspecified arthritis type. The validity of self-reported RA is low (Star et al. 1996). However, because subjects did not know their serum levels of POPs and because their exposure to POPs was mainly due to background exposure, nondifferential misclassification is the most likely consequence of reduced reliability, leading to attenuated strength of association. In this case, null associations could be falsely negative; however, the clear positive associations among women would not be explained by the low validity of RA. In spite of the cross-sectional design, our findings are biologically plausible; this is the first study in the general population with background exposure to POPs. Whether low-dose environmental exposure to POPs in humans could be harmful is one of the most controversial issues in the field of toxicology (Kaiser 2000; Safe 2000; Welshons et al. 2003). However, few epidemiologic studies have been carried out for POPs in the general population, even in a cross-sectional design. The absence of epidemiologic studies in the general population is understandable given the cost of measuring of a variety of POPs and the substantial amount of serum required for their measurement.

Endocrine disruptors such as POPs markedly influence the immune system (Ahmed 2000), but the possibility that the human immune system may respond to a low concentration of POPs has not been studied specifically. However, one might infer such an immune response even to the exposure to the background exposure to POPs on the basis of associations of POPs with diabetes in the general population, which we have reported in this same data set (Lee et al. 2006b). Among the POPs examined in the present study, PCBs were most strongly associated with RA, and the associations did not differ depending on chemical and physical properties of specific PCBs (dioxin-like or nondioxin-like). Interestingly, the known decreasing concentrations of PCBs appear to be consistent with decreasing secular trends of RA over several recent decades in the United States (Alamanos et al. 2006; Doran et al. 2002).

The different associations between PCBs and RA by sex we report here may be biologically plausible because sex hormones appear to play an important role as modulators of autoimmune disease onset and perpetuation, as in the case of RA (Cutolo et al. 2002). Generally, steroid hormones are implicated in the immune response, with estrogens as enhancers at least of the humoral immunity and androgens, progesterone, and glucocorticoids as natural immunosuppressors (Cutolo et al. 2002). Thus, different effects of PCBs by sex may be also possible because endocrine disruptors such as POPs may exert some of their toxicologic effects through sex hormone–related receptors (Crews et al. 2000; Ulbrich and Stahlmann 2004).

Until now, reports of the effects of PCBs on immune function have focused primarily on immunosuppression, with changes in both humoral and cellular immunity (Fernlof et al. 1997; Kimbrough and Krouskas 2001; Lu and Wu 1985; Nakanishi et al. 1985; Svensson et al. 1994; Van Den Heuvel et al. 2002; Weisglas-Kuperus et al. 2000). Studies on specific clinical correlates have suggested increased prevalence of middle ear (Dewailly et al. 2000; Weisglas-Kuperus et al. 2000) and other bacterial infections (Tsuji 2000). Limited information exists on the potential effects of PCBs on autoimmunity. Most epidemiologic studies on autoimmunity have measured cellular or humoral immunologic function, such as numbers in lymphocyte subpopulations, *in vitro* lymphocyte response, plasma cytokine levels, or autoantibodies. However, the results have not been consistent. Although some studies have reported positive associations (Daniel et al. 2001; Langer et al. 2002), other studies failed to do so (Schoenroth et al. 2004; Tsuji 2000; Tsuji et al. 1999; Yu et al. 1998).

In addition to PCBs in the present study, we found that some pesticides were positively associated with RA among women. Previous studies on the association between pesticides and RA have been inconsistent. Several epidemiologic studies have reported increased RA or increased prevalence of antinuclear antibody among farmers (Lundberg et al. 1994; Olsson et al. 2004; Rosenberg et al. 1999), but others did not (De Roos et al. 2005). Interpretation of previous studies may be limited because the exposure assessment was based on questionnaires about exposure to pesticides rather than on direct measurement of pesticide concentrations in blood.

We were able to evaluate the associations of arthritis with a variety of POPs by taking advantage of the NHANES public use data set. In most epidemiologic studies until now, only a limited set of POPs were evaluated. This approach is sensible when the focus is on occupational or accidental exposure to specific POPs. However, in the study of general populations with background exposure to a mixture of POPs, it may be very important to measure a variety of POPs, because the associations of POPs with health outcomes may differ depending on the specific types of POPs in the exposure and the fact that POPs may interact with each other.

Although the strength of association with OA was weaker than with RA, OA still appeared to be associated with some POPs among women. Recently, metabolic factors related to obesity, such as secretion of leptin, have been linked with the onset and progression of OA (Dumond et al. 2003). Thus, POPs may be involved in the pathogenesis of OA because POPs stored in adipose tissue can cause metabolic disturbances, including over-secretion of leptin (Mullerova and Kopecky 2006). Leptin also plays a key role in a host of autoimmune inflammatory conditions such as RA (Otero et al. 2006).

The present study has several limitations. First, the cross-sectional study design in NHANES does not allow inferences to be drawn regarding temporality of events and the causality between POPs and arthritis. Second, misclassification bias is possible because some subjects with a higher POP value but a lower sample volume could be classified in the reference group, or vice versa. Such misclassification would be nondifferential if sample volume is unrelated to the presence of arthritis. Third, as discussed above, the validity of self-reported RA is low (Star et al. 1996) even though it may be also associated with nondifferential misclassification, leading to attenuated strength of association.

In the present study, serum concentrations of both dioxin-like and nondioxin-like PCBs were positively and possibly nonlinearly associated with arthritis among women. The relations were stronger with RA than with OA. These results raise the possibility that background exposures to some POPs may be involved in the pathogenesis of autoimmune diseases such as RA in women. To confirm these relationships, further study in a prospective cohort study would be necessary.

## Figures and Tables

**Table 1:** Age-adjusted Spearman correlation coefficients^a^ between five categories of lipid-adjusted POPs (3 PCDDs, 3 PCDFs, 4 dioxin-like PCBs, 5 nondioxin-like PCBs, and 4 OC pesticides) with demographic or health behavior factors by sex.

Characteristic	PCDDs	PCDFs	Dioxin-like PCBs	Nondioxin-like PCBs	OC pesticides
Males					
Age	0.53**	0.35**	0.65**	0.69**	0.72**
Race	NS	0.07*	NS	NS	−0.29**
Poverty income ratio	NS	NS	0.10**	0.10**	−0.17**
BMI	0.24**	0.14**	0.15**	NS	0.18**
Current smoker	−0.14**	NS	−0.13**	NS	NS
Females					
Age	0.60**	0.52**	0.76**	0.73**	0.78**
Race	−0.10**	0.08*	0.08*	0.10**	−0.35**
Poverty income ratio	NS	0.07*	0.09**	0.12**	−0.16**
BMI	NS	NS	NS	−0.17**	NS
Current smoker	−0.19**	NS	NS	0.09**	NS

NS, not significant. For race, white = 1, and others = 0. For current smoker, current = 1, and others = 0.

a Before calculating correlation coefficients, detectable values of each POP were individually ranked, and the rank order of the individual POPs in each subclass were summed to arrive at the subclass value; all nondetectable values were ranked as 0.

* *p* < 0.05.

** *p* < 0.01.

**Table 2:** Adjusted^a^ OR (95% CI) of prevalence of arthritis by quartiles of PCDDs, PCDFs, dioxin-like PCBs, non-dioxin-like PCBs, and OC pesticides in males and females.^b^

Analyte	< 25th	25th to < 50th	50th to < 75th	≥ 75th	*p*_trend_
Males					
PCDDs					
Cases/participants (no.)	17/191	35/193	47/193	65/192	
Adjusted OR (95% CI)	Referent	1.5 (0.8–2.9)	1.6 (0.8–3.1)	1.4 (0.7–2.8)	0.48
PCDFs					
Cases/participants (no.)	30/192	29/192	43/193	62/192	
Adjusted OR (95% CI)	Referent	0.7 (0.4–1.2)	0.9 (0.5–1.6)	1.1 (0.6–1.9)	0.52
Dioxin-like PCBs					
Cases/participants (no.)	16/192	32/192	47/193	69/192	
Adjusted OR (95% CI)	Referent	1.3 (0.6–2.5)	1.1 (0.6–2.3)	1.3 (0.6–2.8)	0.56
Nondioxin-like PCBs					
Cases/participants (no.)	12/192	24/192	72/193	56/192	
Adjusted OR (95% CI)	Referent	1.2 (0.5–2.6)	3.3 (1.6–6.9)	1.5 (0.7–3.4)	0.23
OC pesticides					
Cases/participants (no.)	14/192	28/192	43/193	62/192	
Adjusted OR (95% CI)	Referent	1.1 (0.5–2.3)	1.5 (0.7–3.2)	1.2 (0.5–2.7)	0.72
Females					
PCDDs					
Cases/participants (no.)	28/238	54/238	62/238	106/238	
Adjusted OR (95% CI)	Referent	1.4 (0.8–2.5)	1.0 (0.6–1.9)	1.3 (0.7–2.4)	0.67
PCDFs					
Cases/participants (no.)	31/237	52/239	63/238	104/238	
Adjusted OR (95% CI)	Referent	1.2 (0.7–2.1)	0.9 (0.5–1.6)	1.3 (0.8–2.3)	0.51
Dioxin-like PCBs					
Cases/participants (no.)	11/237	36/239	91/238	112/238	
Adjusted OR (95% CI)	Referent	2.1 (1.0–4.5)	3.5 (1.7–7.4)	2.9 (1.3–6.5)	0.02
Nondioxin-like PCBs					
Cases/participants (no.)	16/238	34/238	89/238	111/238	
Adjusted OR (95% CI)	Referent	1.6 (0.8–3.1)	2.6 (1.3–5.1)	2.5 (1.2–5.2)	0.02
OC pesticides					
Cases/participants (no.)	19/238	37/238	73/238	121/238	
Adjusted OR (95% CI)	Referent	1.2 (0.6–2.3)	1.3 (0.7–2.6)	1.8 (0.9–3.9)	0.09

a Adjusted for age, race, poverty income ratio, BMI, and cigarette smoking.

b Detectable values of each POP were individually ranked, and the rank orders of the individual POPs in each subclass were summed to arrive at the subclass value; all nondetectable values were ranked as 0.

**Table 3:** Adjusted^a^ OR (95% CI) of prevalence of arthritis by categories of specific POPs belonging to dioxin-like PCBs, nondioxin-like PCBs, and OC pesticides in females.

		Detectable^b^				
Analyte	Nondetectable	< 25th	25th to < 50th	50th to < 75th	≥ 75th	*p*_trend_
Dioxin-like PCBs						
2,4,4′,5-Tetrachlorobiphenyl (PCB-74)						
Cases/participants (no.)	20/334	26/151	53/158	7/154	81/155	
Adjusted OR (95% CI)	Referent	2.1 (1.1–4.1)	2.9 (1.5–5.6)	3.4 (1.8–6.7)	3.2 (1.5–6.7)	< 0.01
2,3′,4,4′,5-Pentachlorobiphenyl (PCB-118)						
Cases/participants (no.)	21/306	27/162	47/161	74/161	81/162	
Adjusted OR (95% CI)	Referent	1.6 (0.8–3.1)	2.1 (1.1–3.9)	3.0 (1.6–5.8)	2.2 (1.1–4.5)	0.01
3,3′,4,4′,5-Pentachlorobiphenyl (PCB-126)						
Cases/participants (no.)	26/174	25/193	32/195	70/196	97/194	
Adjusted OR (95% CI)	Referent	0.9 (0.4–1.6)	0.7 (0.4–1.3)	1.3 (0.7–2.3)	1.4 (0.8–2.7)	0.07
3,3′,4,4′,5,5′-Hexachlorobiphenyl (PCB-169)						
Cases/participants (no.)	26/221	15/183	47/183	76/183	86/182	
Adjusted OR (95% CI)	Referent	0.7 (0.3–1.4)	1.4 (0.8–2.6)	1.6 (0.9–3.0)	1.6 (0.8–3.1)	0.08
Nondioxin-like PCBs						
2,2′,3,4,4′,5-Hexachlorobiphenyl (PCB-138)						
Cases/participants (no.)	26/233	17/180	52/179	73/181	82/179	
Adjusted OR (95% CI)	Referent	0.9 (0.4–1.8)	1.6 (0.9–2.9)	1.7 (1.0–3.1)	1.3 (0.7–2.5)	0.21
2,2′,4,4′,5,5′-Hexachlorobiphenyl (PCB-153)						
Cases/participants (no.)	20/199	12/188	50/188	83/189	85/188	
Adjusted OR (95% CI)	Referent	0.7 (0.3–1.5)	1.7 (0.9–3.3)	2.3 (1.2–4.4)	1.6 (0.8–3.2)	0.06
2,2′,3,3′,4,4′,5-Heptachlorobiphenyl (PCB-170)						
Cases/participants (no.)	24/344	36/152	47/152	70/152	73/152	
Adjusted OR (95% CI)	Referent	2.9 (1.6–5.3)	1.9 (1.0–3.6)	2.9 (1.4–5.6)	2.7 (1.3–5.6)	0.03
2,2′,3,4,4′,5,5′-Heptachlorobiphenyl (PCB-180)						
Cases/participants (no.)	16/234	21/179	48/179	76/181	89/179	
Adjusted OR (95% CI)	Referent	1.4 (0.7–2.9)	2.1 (1.1–4.2)	2.7 (1.3–5.7)	2.7 (1.2–6.0)	< 0.01
2,2′,3,4′,5,5′,6-Heptachlorobiphenyl (PCB-187)						
Cases/participants (no.)	33/396	35/137	46/141	67/140	69/138	
Adjusted OR (95% CI)	Referent	1.9 (1.1–3.3)	1.9 (1.0–3.4)	2.8 (1.5–5.1)	2.2 (1.1–4.3)	0.01
OC pesticides						
* p,p*′-Dichlorodiphenyldichloroethane						
Cases/participants (no.)	0/0	31/236	44/240	84/239	91/237	
Adjusted OR (95% CI)		Referent	0.9 (0.5–1.6)	1.1 (0.6–2.0)	1.0 (0.5–1.8)	0.93
Oxychlordane						
Cases/participants (no.)	11/185	17/191	51/192	67/192	104/192	
Adjusted OR (95% CI)	Referent	1.1 (0.5–2.6)	2.6 (1.2–5.4)	2.0 (0.9–4.5)	3.1 (1.3–7.1)	< 0.01
* trans*-Nonachlor						
Cases/participants (no.)	5/104	20/210	47/215	75/212	103/211	
Adjusted OR (95% CI)	Referent	1.4 (0.5–4.0)	2.0 (0.7–5.6)	2.4 (0.9–6.8)	2.2 (0.8–6.6)	0.07
β-Hexachlorocyclohexane						
Cases/participants (no.)	17/225	24/181	58/182	74/182	77/182	
Adjusted OR (95% CI)	Referent	1.0 (0.5–1.9)	1.7 (0.8–3.4)	1.5 (0.7–3.1)	1.4 (0.6–3.2)	0.37

a Adjusted for age, race, poverty income ratio, BMI, and cigarette smoking.

b For each POP, subjects with serum concentrations < LOD were regarded as the reference group.

**Table 4:** Adjusted^a^ OR (95% CI) of prevalence of RA, OA, or unspecified arthritis by categories of dioxin-like PCBs, nondioxin-like PCBs, or OC pesticides in females.^b^

Outcome/analyte	< 25th	25th to < 50th	50th to < 75th	≥ 75th	*p*_trend_
RA					
Dioxin-like PCBs					
Cases/participants (no.)	2/228	16/219	17/164	26/152	
Adjusted OR (95% CI)	Referent	7.6 (1.7–34.4)	6.1 (1.2–29.7)	8.5 (1.6–44.5)	0.05
Nondioxin-like PCBs					
Cases/participants (no.)	4/226	9/213	21/710	27/154	
Adjusted OR (95% CI)	Referent	2.2 (0.6–7.4)	4.4 (1.3–15.2)	5.4 (1.4–20.3)	< 0.01
OC pesticides					
Cases/participants (no.)	4/223	13/214	19/184	25/142	
Adjusted OR (95% CI)	Referent	2.8 (0.8–8.9)	2.7 (0.7–9.8)	3.5 (0.9–14.0)	0.15
OA					
Dioxin-like PCBs					
Cases/participants (no.)	3/229	12/215	28/175	33/159	
Adjusted OR (95% CI)	Referent	1.9 (0.5–7.2)	2.0 (0.5–7.6)	1.6 (0.4–6.4)	0.97
Nondioxin-like PCBs					
Cases/participants (no.)	4/226	12/216	30/179	30/157	
Adjusted OR (95% CI)	Referent	1.6 (0.5–5.3)	1.7 (0.5–5.8)	1.2 (0.3–4.5)	0.90
OC pesticides					
Cases/participants (no.)	4/223	12/213	22/187	38/155	
Adjusted OR (95% CI)	Referent	1.4 (0.4–4.6)	1.3 (0.4–5.2)	2.1 (0.6–8.2)	0.21
Unspecified arthritis					
Dioxin-like PCBs					
Cases/participants (no.)	5/231	8/211	38/185	44/170	
Adjusted OR (95% CI)	Referent	1.2 (0.4–3.9)	3.8 (1.3–11.2)	2.9 (0.9–9.3)	0.06
Nondioxin-like PCBs					
Cases/participants (no.)	7/229	9/213	36/185	43/170	
Adjusted OR (95% CI)	Referent	1.2 (0.4–3.3)	2.8 (1.0–7.4)	2.8 (1.0–8.3)	0.03
OC pesticides					
Cases/participants (no.)	8/228	10/209	24/188	53/172	
Adjusted OR (95% CI)	Referent	0.8 (0.3–2.3)	1.0 (0.4–2.8)	1.6 (0.6–4.6)	0.14

a Adjusted for age, race, poverty income ratio, BMI, and cigarette smoking.

b Detectable values of each POP were individually ranked, and the rank orders of the individual POPs in each subclass were summed to arrive at the subclass value; all nondetectable values were ranked as 0.

## References

[b1-ehp0115-000883] Abelsohn A, Gibson BL, Sanborn MD, Weir E (2002). Identifying and managing adverse environmental health effects: 5. Persistent organic pollutants. CMAJ.

[b2-ehp0115-000883] Abyad A, Boyer JT (1992). Arthritis and aging. Curr Opin Rheumatol.

[b3-ehp0115-000883] Ahmed SA (2000). The immune system as a potential target for environmental estrogens (endocrine disrupters): a new emerging field. Toxicology.

[b4-ehp0115-000883] Alamanos Y, Voulgari PV, Drosos AA (2006). Incidence and prevalence of rheumatoid arthritis, based on the 1987 American College of Rheumatology criteria: a systematic review. Semin Arthritis Rheum.

[b5-ehp0115-000883] Chevrier J, Dewailly E, Ayotte P, Mauriege P, Despres JP, Tremblay A (2000). Body weight loss increases plasma and adipose tissue concentrations of potentially toxic pollutants in obese individuals. Int J Obes Relat Metab Disord.

[b6-ehp0115-000883] Crews D, Willingham E, Skipper JK (2000). Endocrine disruptors: present issues, future directions. Q Rev Biol.

[b7-ehp0115-000883] Cutolo M, Villaggio B, Craviotto C, Pizzorni C, Seriolo B, Sulli A (2002). Sex hormones and rheumatoid arthritis. Autoimmun Rev.

[b8-ehp0115-000883] Daniel V, Huber W, Bauer K, Suesal C, Conradt C, Opelz G (2001). Associations of blood levels of PCB, HCHS, and HCB with numbers of lymphocyte subpopulations, i*n vitro* lymphocyte response, plasma cytokine levels, and immunoglobulin autoantibodies. Environ Health Perspect.

[b9-ehp0115-000883] De Roos AJ, Cooper GS, Alavanja MC, Sandler DP (2005). Rheumatoid arthritis among women in the Agricultural Health Study: risk associated with farming activities and exposures. Ann Epidemiol.

[b10-ehp0115-000883] Dewailly E, Ayotte P, Bruneau S, Gingras S, Belles-Isles M, Roy R (2000). Susceptibility to infections and immune status in Inuit infants exposed to organochlorines. Environ Health Perspect.

[b11-ehp0115-000883] Doran MF, Pond GR, Crowson CS, O’Fallon WM, Gabriel SE (2002). Trends in incidence and mortality in rheumatoid arthritis in Rochester, Minnesota, over a forty-year period. Arthritis Rheum.

[b12-ehp0115-000883] Dumond H, Presle N, Terlain B, Mainard D, Loeuille D, Netter P (2003). Evidence for a key role of leptin in osteoarthritis. Arthritis Rheum.

[b13-ehp0115-000883] Fernlof G, Gadhasson I, Podra K, Darnerud PO, Thuvander A (1997). Lack of effects of some individual polybrominated diphenyl ether (PBDE) and polychlorinated biphenyl (PCB) congeners on human lymphocyte functions in vitro. Toxicol Lett.

[b14-ehp0115-000883] Gabriel SE (2001). The epidemiology of rheumatoid arthritis. Rheum Dis Clin North Am.

[b15-ehp0115-000883] Graubard BI, Korn EL (1999). Analyzing health surveys for cancer-related objectives. J Natl Cancer Inst.

[b16-ehp0115-000883] Kaiser J (2000). Endocrine disrupters. Panel cautiously confirms low-dose effects. Science.

[b17-ehp0115-000883] Kimbrough RD, Krouskas CA (2001). Polychlorinated biphenyls, dibenzo-*p*-dioxins, and dibenzofurans and birth weight and immune and thyroid function in children. Regul Toxicol Pharmacol.

[b18-ehp0115-000883] Korn EL, Graubard BI (1991). Epidemiologic studies utilizing surveys: accounting for the sampling design. Am J Public Health.

[b19-ehp0115-000883] Langer P, Tajtakova M, Guretzki HJ, Kocan A, Petrik J, Chovancova J (2002). High prevalence of anti-glutamic acid decarboxylase (anti-GAD) antibodies in employees at a polychlorinated biphenyl production factory. Arch Environ Health.

[b20-ehp0115-000883] Lee DH, Jacobs DR, Porta M (2006a). Could low level background exposure to persistent organic pollutants contribute to the social burden of type 2 diabetes?. [Editorial] J Epidemiol Community Health.

[b21-ehp0115-000883] Lee DH, Lee IK, Song KE, Steffes M, Toscano W, Baker BA (2006b). A strong dose-response relation between serum concentrations of persistent organic pollutants and diabetes: results from the National Health and Examination Survey. Diabetes Care.

[b22-ehp0115-000883] Lu YC, Wu YC (1985). Clinical findings and immunological abnormalities in Yu-Cheng patients. Environ Health Perspect.

[b23-ehp0115-000883] Lundberg I, Alfredsson L, Plato N, Sverdrup B, Klareskog L, Kleinau S (1994). Occupation, occupational exposure to chemicals and rheumatological disease. A register based cohort study. Scand J Rheumatol.

[b24-ehp0115-000883] Mullerova D Kopecky J 2006. White adipose tissue: storage and effector site for environmental pollutants. Physiol Res. Available: http://www.biomed.cas.cz/physiolres/pdf/prepress/1022.pdf [accessed 17 April 2007]. 10.33549/physiolres.931022 16925464

[b25-ehp0115-000883] Nakanishi Y, Shigematsu N, Kurita Y, Matsuba K, Kanegae H, Ishimaru S (1985). Respiratory involvement and immune status in Yusho patients. Environ Health Perspect.

[b26-ehp0115-000883] NCHS 2005. Third National Report on Human Exposure to Environmental Chemicals. Atlanta, GA:National Center for Health Statistics. Available: http://www.cdc.gov/exposurereport/pdf/thirdreport.pdf [accessed 20 October 2006].

[b27-ehp0115-000883] NCHS (National Center for Health Statistics) 2006a. National Health and Nutrition Examination Survey: NHANES 1999–2000. Available: http://www.cdc.gov/nchs/about/major/nhanes/nhanes99_00.htm [accessed 20 October 2006].

[b28-ehp0115-000883] NCHS (National Center for Health Statistics) 2006b. National Health and Nutrition Examination Survey: NHANES 2000–2001. Available: http://www.cdc.gov/nchs/about/major/nhanes/nhanes01-02.htm [accessed 20 October 2006].

[b29-ehp0115-000883] Olsson AR, Skogh T, Axelson O, Wingren G (2004). Occupations and exposures in the work environment as determinants for rheumatoid arthritis. Occup Environ Med.

[b30-ehp0115-000883] Otero M, Lago R, Gomez R, Lago F, Dieguez C, Gomez-Reino JJ (2006). Changes in plasma levels of fat-derived hormones adiponectin, leptin, resistin and visfatin in patients with rheumatoid arthritis. Ann Rheum Dis.

[b31-ehp0115-000883] Rosenberg AM, Semchuk KM, McDuffie HH, Ledingham DL, Cordeiro DM, Cessna AJ (1999). Prevalence of antinuclear antibodies in a rural population. J Toxicol Environ Health A.

[b32-ehp0115-000883] Safe SH (2000). Endocrine disruptors and human health—is there a problem? An update. Environ Health Perspect.

[b33-ehp0115-000883] Schoenroth L, Chan S, Fritzler M (2004). Autoantibodies and levels of polychlorinated biphenyls in persons living near a hazardous waste treatment facility. J Investig Med.

[b34-ehp0115-000883] Sharma L, Kapoor D, Issa S (2006). Epidemiology of osteoarthritis: an update. Curr Opin Rheumatol.

[b35-ehp0115-000883] Star VL, Scott JC, Sherwin R, Lane N, Nevitt MC, Hochberg MC (1996). Validity of self-reported rheumatoid arthritis in elderly women. J Rheumatol.

[b36-ehp0115-000883] Svensson BG, Hallberg T, Nilsson A, Schutz A, Hagmar L (1994). Parameters of immunological competence in subjects with high consumption of fish contaminated with persistent organochlorine compounds. Int Arch Occup Environ Health.

[b37-ehp0115-000883] Tsuji H (2000). Immune effects of endocrine disruptors. Nippon Rinsho.

[b38-ehp0115-000883] Tsuji H, Hirahashi T, Ogata H, Fujishima M (1999). Serum immunoglobulin concentrations and autoantibodies in patients with Yusho. Fukuoka Igaku Zasshi.

[b39-ehp0115-000883] Turner W, DiPietro E, Lapeza C, Green V, Gill J, Patterson DG (1997). A fast universal automated cleanup system for the isotope-dilution HRMS analysis of PCDDs, PCDFs, coplanar PCBs, PCB congeners, and persistent pesticides from the same serum sample. Organohalogen Compounds.

[b40-ehp0115-000883] Ulbrich B, Stahlmann R (2004). Developmental toxicity of poly-chlorinated biphenyls (PCBs): a systematic review of experimental data. Arch Toxicol.

[b41-ehp0115-000883] Van Den Heuvel RL, Koppen G, Staessen JA, Hond ED, Verheyen G, Nawrot TS (2002). Immunologic bio-markers in relation to exposure markers of PCBs and dioxins in Flemish adolescents (Belgium). Environ Health Perspect.

[b42-ehp0115-000883] Weisglas-Kuperus N, Patandin S, Berbers GA, Sas TC, Mulder PG, Sauer PJ (2000). Immunologic effects of background exposure to polychlorinated biphenyls and dioxins in Dutch preschool children. Environ Health Perspect.

[b43-ehp0115-000883] Welshons WV, Thayer KA, Judy BM, Taylor JA, Curran EM, vom Saal FS (2003). Large effects from small exposures. I. Mechanisms for endocrine-disrupting chemicals with estrogenic activity. Environ Health Perspect.

[b44-ehp0115-000883] Yu ML, Hsin JW, Hsu CC, Chan WC, Guo YL (1998). The immunologic evaluation of the Yucheng children. Chemosphere.

